# Effects of plant species diversity on nematode community composition and diversity in a long-term biodiversity experiment

**DOI:** 10.1007/s00442-021-04956-1

**Published:** 2021-06-06

**Authors:** Peter Dietrich, Simone Cesarz, Tao Liu, Christiane Roscher, Nico Eisenhauer

**Affiliations:** 1 Department of Physiological Diversity, UFZ, Helmholtz Centre for Environmental Research, Permoser Straße 15, 04318 Leipzig, Germany; 2 German Centre of Integrative Biodiversity Research (iDiv) Halle-Jena-Leipzig, Puschstraße 4, 04103 Leipzig, Germany; 3 Department of Experimental Interaction Ecology, Institute of Biology, Leipzig University, Puschstraße 4, 04103 Leipzig, Germany; 4 Key Laboratory of Vegetation Restoration and Management of Degraded Ecosystems, South China Botanical Garden, Chinese Academy of Sciences, Guangzhou, 510650 China

**Keywords:** Aboveground–belowground interactions, Biodiversity loss, Plant–soil interactions, Resource quality, Resource quantity

## Abstract

Diversity loss has been shown to change the soil community; however, little is known about long-term consequences and underlying mechanisms. Here, we investigated how nematode communities are affected by plant species richness and whether this is driven by resource quantity or quality in 15-year-old plant communities of a long-term grassland biodiversity experiment. We extracted nematodes from 93 experimental plots differing in plant species richness, and measured above- and belowground plant biomass production and soil organic carbon concentrations (*C*_org_) as proxies for resource quantity, as well as *C*/*N*_leaf_ ratio and specific root length (SRL) as proxies for resource quality. We found that nematode community composition and diversity significantly differed among plant species richness levels. This was mostly due to positive plant diversity effects on the abundance and genus richness of bacterial-feeding, omnivorous, and predatory nematodes, which benefited from higher shoot mass and soil *C*_org_ in species-rich plant communities, suggesting control via resource quantity. In contrast, plant-feeding nematodes were negatively influenced by shoot mass, probably due to higher top–down control by predators, and were positively related to SRL and *C*/*N*_leaf_, indicating control via resource quality. The decrease of the grazing pressure ratio (plant feeders per root mass) with plant species richness indicated a higher accumulation of plant-feeding nematodes in species-poor plant communities. Our results, therefore, support the hypothesis that soil-borne pathogens accumulate in low-diversity communities over time, while soil mutualists (bacterial-feeding, omnivorous, predatory nematodes) increase in abundance and richness in high-diversity plant communities, which may contribute to the widely-observed positive plant diversity–productivity relationship.

**Supplementary Information:**

The online version contains supplementary material available at 10.1007/s00442-021-04956-1.

## Introduction

The relationship between plants and soil biota is important for controlling plant productivity, decomposition, and nutrient cycling (Neher 2010; van der Heijden et al. 2008). Increasing intensity of global change impacts and subsequent biodiversity loss might, however, disrupt this relationship (Classen et al. 2015; Eisenhauer 2012), with unknown consequences for the ecosystems and the well-being of humans (FAO et al. 2020; Wall et al. 2015). Therefore, it is essential to fully understand the underlying mechanisms of the complex relationships between plants and soil communities to better assess the future impact of global change.

There is an overwhelming diversity of organisms living in the soil. Only one gram of soil can contain thousands of species of bacteria, fungi, protozoa, and nematodes (FAO et al. 2020). Drawing mechanistic links between plant species diversity and soil organisms by investigating each soil community member are challenging. Therefore, it is a frequent practice in ecological research to focus on key groups, which act as indicators of soil biodiversity and health. One of the most important groups of soil organisms are nematodes because they are the most abundant Metazoan in soil (van den Hoogen et al. 2019), they occupy all trophic levels and thus play a key role in the soil food web (Yeates et al. 1993). The most studied trophic group are plant feeding-nematodes, best-known as pests in agricultural systems (Neher 2010), and one of the soil biota groups that are suspected to be responsible for productivity loss over time in species-poor plant communities (Eisenhauer et al. 2012). Next to these, there are bacterial feeders, fungal feeders, predators, and omnivores, which can be beneficial for plants due to the increase of microbial activity and soil nutrients (due to microbivores) and suppression of pests due to top–down control (caused by omnivores and predators) (Wilschut and Geisen 2021). In addition to this information about the structure and topology of the food web, nematodes can also be used as bioindicators of ecosystem conditions and health (Wilschut and Geisen 2021). For example, frequent disturbances can be indicated by many small, fast-growing nematodes, while large, slow-growing nematodes would be absent in these systems. Furthermore, nematode community indices can provide information about environmental conditions, ecosystem functioning in the soil, pollutants, and whether the system is more dominated by bacteria or fungi (Wilschut and Geisen 2021).

Due to the plethora of information that nematodes can offer, they have been investigated in several biodiversity experiments to determine the effect of plant species loss on plant–soil interactions, soil food webs, and soil ecosystem processes (Cesarz et al. 2017; Cortois et al. 2017; De Deyn et al. 2004; Eisenhauer et al. 2011, 2013; Viketoft et al. 2009, 2011). These studies have shown that there is a strong impact of plant community composition and diversity on the nematode community; however, the underlying mechanisms are not fully clarified (Wilschut and Geisen 2021) and plant diversity effects may change over time (Eisenhauer et al. 2012; Reich et al. 2012). In the present study, we aimed to address two knowledge gaps, which are highly important to understand previous inconsistent results and underlying mechanisms. First, there is a lack of studies investigating the long-term consequences of plant diversity loss on nematodes. This is highly relevant because differences in nematode communities among plant species richness levels increase over time (Sohlenius et al. 2011; Viketoft et al. 2011), indicating time-lags of nematode responses to changes in plant diversity (Eisenhauer et al. 2012). Most of the recent investigations were after a relatively short duration of the biodiversity experiment (≤ 8 years). To our knowledge, there were only a few studies investigating communities older than ten years (Bennett et al. 2020; Cesarz et al. 2017; Eisenhauer et al. 2013); however, in these longer-term studies, the focus was more on the nematode response to disturbances and/or interaction effects with other environmental changes. Second, there is a lack of studies investigating in more detail the underlying mechanisms responsible for the effects of plant diversity loss on nematodes (Cortois et al. 2017). A small number of studies investigated the impact of resource quantity (e.g., increased biomass in species-rich communities) on nematodes, with inconsistent results (Cortois et al. 2017; Eisenhauer et al. 2013; Viketoft et al. 2009). However, only one study (Cortois et al. 2017), to our knowledge, also tested the impact of resource quality on nematode responses in a biodiversity experiment.

In the present study, we tried to fill these two knowledge gaps by investigating the composition and diversity of nematodes in 15-year-old plant communities of a long-term biodiversity experiment (Jena Experiment; Roscher et al. 2004). We explored how nematode communities were influenced by plant species richness after 15 years and whether this is driven by changes in the quantity or quality of resources using structural equation modeling. For this, we sampled nematodes in 93 experimental plots differing in plant species richness from one to nine species. Moreover, we sampled roots and shoots to assess biomass production from these plots and measured soil organic carbon as proxies for resource quantity. As proxies for resource quality, we determined leaf *C*/*N* ratios and root traits (specific root length, root length density) of the plant communities.

Due to the experiment's long duration, we expected significant differences in the nematode community composition and diversity among the plant species richness levels. Based on previous knowledge, we further expected higher plant productivity and nutritional quality in species-rich plant communities (due to increased likelihood of including nutrient-rich plant species), which we predicted to be directly related to plant feeders and indirectly to microbial feeders via increased microbial biomass, indicating strong bottom–up effects on these trophic groups (Eisenhauer et al. 2013). We did not assume a direct influence of plant species richness on omnivores and predators, but an indirect effect via an increased number of nematodes as prey (Scherber et al. 2010).

## Materials and methods

### Study site and plot selection

The Jena Experiment is a long-term biodiversity experiment located in the floodplain of the Saale River near the city of Jena (Thuringia, Germany, 50° 55′ N, 11° 35′ E, 130 m a.s.l.; Roscher et al. 2004). Before the experiment was established in 2002, the site had been used as a high-fertilized arable field for growing wheat and vegetables from the early 1960s until 2000 and was kept fallow during the year 2001. Mean annual air temperature for the decade before the present study (2007–2016) was 9.7 °C, and mean annual precipitation was 587 mm, both recorded with an automatic meteorological station at the experimental site (Weather Station Jena-Saaleaue, Max-Planck-Institute for Biogeochemistry Jena, https://www.bgc-jena.mpg.de/wetter/). The soil is a Eutric Fluvisol. The soil texture changes from sandy loam to silty clay with increasing distance from the river Saale. Thus, the experimental area was divided into four blocks, arranged in parallel with increasing distance to the river to account for changes in soil texture (Roscher et al. 2004).

For our study, we used the study plots of a sub-experiment of the Jena Experiment, the so-called Dominance Experiment. This experiment included nine plant species, which often reach dominance in Central European mesophilic grasslands of the Arrhenatherion type (Ellenberg 1988): five grasses (*Alopecurus pratensis* L., *Arrhenatherum elatius* (L.) P. Beauv. ex J. Presl et C. Presl, *Dactylis glomerata* L., *Phleum pratense* L., *Poa trivialis* L.), two legumes (*Trifolium pratense* L., *Trifolium repens* L.), and two herbs (*Anthriscus sylvestris* (L.) Hoffm., *Geranium pratense* L.). Species were grown in plots of 3.5 × 3.5 m from 2002 to 2009 until plot size was reduced to 1 × 1 m (2010–2018). Species richness levels ranged from one to nine species (1, 2, 3, 4, 6, and 9 plant species). Every species and every species pair occurred with the same frequency at each particular species richness level. Each of the monocultures and mixtures were replicated (all *N* = 2; except for the nine-species mixtures [*N* = 8]). The same number of plant communities per species richness level was represented in each of the four blocks, while replicates with identical species compositions were located in different blocks. Seeds from all species were purchased from a commercial supplier (Rieger-Hoffman GmbH, Blaufelden-Raboldshause, Germany) and were sown in May 2002 with a density of 1000 viable seeds per m^2^. Study plots were mown every year in June and September (mown plant material was removed), were regularly weeded to maintain the sown species compositions, and never fertilized.

For our study, we used the 1-, 2-, 6-, and 9-species plots of the Dominance Experiment to keep the number of samples and analyses manageable. Furthermore, two plant species, the grass *P. pratense* and the herb *A. sylvestris*, were not used as target plant species in this study because of their very low abundance in the communities (almost extinct). For the other seven species, we sampled both monocultures and all existing six-species and nine-species communities (Table 1). Moreover, we sampled all existing two-species combinations of the seven species (both replicates), and one replicate with *A. sylvestris* and *P. pratense*, respectively (Supplementary material Table S1). Exceptions were the legumes *T. pratense* and *T. repens*, which were absent in many two-species combinations in 2017. Therefore, we used only one replicate with *A. elatius*, *A. pratensis*, and *D. glomerata* and only one replicate, where both legumes were present (i.e., we chose the replicate with a higher abundance of the legumes; Table S1). Furthermore, for *T. pratense* we used both replicates with *A. sylvestris* (high abundance of *T. pratense*). However, we did not sample the *T. repens–A. sylvestris* combination as well as the two-species communities with *G. pratense* and *A. sylvestris* or *P. pratense*, respectively, because both target species were extinct in these plots (Table S1). In total, we sampled 93 plots (Table 1). Realized plant species richness, i.e., how many target plant species were growing in the plots, was determined in May 2017.

**Table 1:** Summary of the presence of target plant species in monoculture and mixture communities and the total number of plant communities, which were used for the soil and plant sampling

	Plot type			
	Monocultures	Two species	Six species	Nine species
Grass species				
* A. elatius*	2	12	16	8
* A. pratensis*	2	12	16	8
* D. glomerata*	2	12	16	8
* P. trivialis*	2	14	16	8
Herb species				
* G. pratense*	2	12	16	8
Legume species				
* T. pratense*	2	11	16	8
* T. repens*	2	9	16	8
Total no. of communities	14	47	24	8

The numbers (in the species rows) indicate how often a plant species occurred in the listed plot type

### Nematode extraction and identification

Nematodes were sampled on 16 May 2017, i.e., shortly before peak plant biomass. It was a sunny day without rainfall and a maximum temperature of 23.6 °C. Spring (March–May) of 2017 was warmer (10.1 °C) than the average for 2007–2016 (9.2 °C) and had a slightly higher amount of precipitation (average: 138.29 mm, 2017: 150.59 mm; Weather Station Jena-Saaleaue, Max-Planck-Institute for Biogeochemistry Jena). On each of the 93 plots, three randomly located soil cores (2.5 cm diameter) were taken to a depth of 10 cm. Samples were kept in plastic bags, transported to the laboratory in a cooling box, and stored in a fridge at 4 °C. Soil cores per plot were pooled and sieved at 4 mm to remove stones and roots. A subsample of 25 g of sieved soil was taken for the extraction of free-living nematodes using a modified Baermann method (Cesarz et al. 2019; Ruess 1995). The subsamples were filled in plastic pots with a bottom consisting of gauze and milk filter and placed in funnels extended by a plastic tube that was closed with a clamp. Funnels were then filled with tap water up to the bottom of the pots and kept for 72 h at room temperature (~ 20 °C) for nematode migration from soil to water. After that, water was filtered through a sieve with a 15 µm mesh to separate nematodes from water. Nematodes were transferred into vials (by rinsing the mesh) and killed and fixed in formaldehyde solution (4%). The remaining soil samples were dried at 45 °C for 72 h and weighed to calculate the number of nematodes per 100 g dry soil. Animals were counted per sample, and 100 individuals (or up to 100 individuals, when less than 100 were present) were identified to genus level (Bongers 1988) using 400 × magnification (microscope DMI4000 B, Leica, Wetzlar, Germany).

### Calculation of nematode indices

Nematodes were extrapolated to 100 g dry soil, and genus richness and Shannon–Wiener diversity based on genera (= nematode diversity) were calculated (Neher and Darby 2009). To identify changes in the food web structure, nematodes were divided into the trophic groups plant feeders (PF), fungal feeders (FF), bacterial feeders (BF), predators (Pr), and omnivores (Om), according to Yeates et al. (1993); except the genus *Filenchus*, which was assigned to fungal feeders (Okada et al. 2005). We determined the total number of nematodes and the number of genera within each trophic group. Furthermore, we calculated three ratios based on trophic group classification: the predator–prey ratio (Pr + Om/PF), indicating the ability of the nematode community to prevent accumulation of plant feeders (top–down control); the ratio between fungal and bacterial feeders (FF/(FF + BF)), indicating changes in the relevance of energy channels (= channel ratio; Neher and Darby 2009); and the log ratio of plant feeders to root mass (log(PF/root mass)), indicating nematode-induced grazing pressure (= grazing pressure ratio; Cortois et al. 2017). We further divided the nematodes according to their *r* vs. *K* life-history strategy using the colonizer (c)–persister (p) scale, which ranges from 1 to 5 (Bongers and Bongers 1998; Ferris et al. 2001). Nematodes within the same c–p group show similar responses to changes in their environment. Responses range from extreme *r*-strategists (c–p1 = short life cycle, high fecundity, tolerant to disturbance) to extreme *K*-strategists (c–p 5 = long life cycle, produce few large eggs, long generation time, sensitive to disturbance; Bongers and Bongers 1998). Plant feeders were excluded from these calculations, as they react differently to nutrient availability (Bongers and Bongers 1998). Using the c–p scale and the trophic classification, we calculated three indices: the Structure Index (SI), indicating the structure and complexity of the soil food web (Ferris et al. 2001); the Enrichment Index (EI), indicating the nutrient status of the soil system (Ferris et al. 2001); and the Maturity Index (MI), which shows the overall soil food-web complexity and disturbance level (Bongers and Bongers 1998; Neher and Darby 2009). Furthermore, we grouped the c–p 1 and c–p 2 nematodes as classic *r*-strategists, while the c–p 3, c–p 4, and c–p 5 nematodes represent *K*-strategists with increasing intensity.

### Plant and soil variables

To test the impact of plant community properties on the nematode community, we measured plant community biomass (root and shoot mass) as a proxy for resource quantity and *C*/*N*_leaf_ and root traits (specific root length, root length density) as proxies for resource quality. We used leaf rather than root samples for our *C*/*N* analyses because *C*/*N*_leaf_ better represents heterogeneity within the community (leaves were collected from different individuals per species all around the plot), while root *C*/*N* could only have been measured from the root biomass of two soil cores. Due to the small size of the plots, we were only able to take a limited number of soil cores without permanently disturbing the plots. Nevertheless, previous work showed a positive correlation between leaf and root nitrogen concentration (Siebenkäs et al. 2015; Simpson et al. 2020), as well as *C*/*N*_leaf_ and *C*/*N*_root_ (Ferlian et al. 2017; Legay et al. 2016) making *C*/*N*_leaf_ a useful predictor for resource quality.

Shoot mass was harvested block-wise on each plot from 29 May to 5 June 2017. A sample area of 0.2 × 0.5 m was chosen in the center of the plots, excluding the outer margin (0.25 m), and plants were cut five 5 cm above ground level. Biomass samples were sorted (target plant species, weed, dead plant material), dried at 70 °C for 48 h, and weighed. Shoot mass per plot and species were extrapolated to one square meter (g_shoot_ m^−2^). Shortly before the harvest, we sampled 10 to 15 fully developed leaves from all plant species on all plots, if possible (species by species and block-wise). Leaf samples per species and plot were dried for 48 h at 70 °C and ground to fine powder with a mixer mill (MM2000, Retsch, Haan, Germany). Milled plant material (10 mg) was used to determine leaf carbon and nitrogen concentrations (mg *N* g_leaf_^−1^, mg *C* g_leaf_^−1^) with an elemental analyzer (Vario EL Element Analyzer, Elementar, Hanau, Germany) and to derive *C*/*N*_leaf_ ratios per plot and species. Using these values, we calculated the community-weighted mean (CWM) per plot as the mean trait value weighted by species relative abundances according to the equation:

$${\text{CWM}}_{C/N} = \mathop \sum \limits_{i = 1}^{S} p_{i } t_{i} ,$$

where *S* is the number of species in the community, *p*_*i*_ are the species biomass proportions, and *t*_*i*_ are species-specific *C*/*N*_leaf_ values.

For root-related measurements, we took two soil cores (10 cm depth, 5 cm diameter) per plot on 20 and 21 June 2017. The sampling location for each soil core was randomly selected in the inner area of the plot (at least 25 cm distance from the edges). For the monoculture plots, we only sampled one replicate per plant species to keep the disturbance as low as possible. Soil cores were pooled per plot and stored in a freezer (− 20 °C). At a later point, soil cores were defrosted, and roots were cleaned by rinsing off all soil over a 0.5 mm sieve. To determine root traits, root samples per plot were scanned on a flatbed scanner at 800 dpi directly after cleaning (Epson Expression 10,000 XL scanner, Regent Instruments, Quebec, Canada), and root length was measured with an image analysis software (WinRHIZO; Regent Instruments, Quebec City, Canada). After that, roots were dried for 48 h at 70 °C and weighed (= root mass, g_root_ cm_soil_^−3^). Specific root length (= SRL) was calculated as the ratio of root length to root mass (m_root_ g_root_^−1^) and root length density (= RLD) as the ratio of root length to the soil volume of both soil cores per plot (cm_root_ cm_soil_^−3^).

We also determined soil organic carbon and soil nitrogen concentrations to test whether these soil variables have an impact on the nematode community. Both variables have been shown to be strongly related to the soil microbial community (Lange et al. 2015; Prommer et al. 2020), and were thus classified as additional proxies for resource quantity. However, because of the strong positive correlation between organic carbon and nitrogen (*r* = 0.866, *P* < 0.001), we decided to only use organic carbon for statistical analyses. For determination, we took subsamples from the freshly sieved soil (4 mm) for the nematode extraction, which was further sieved to 2 mm and then air-dried. After this, the soil samples were ground to a fine powder with a mixer mill (MM2000, Retsch, Haan, Germany) and dried at 40 °C for 5 h. Soil nitrogen and total carbon concentrations were analyzed with an elemental analyzer (Vario EL Element Analyzer, Elementar, Hanau, Germany) using 40 mg of the ground soil. To determine the concentration of soil organic carbon, we measured the concentrations of soil carbonate volumetrically with a calcimeter according to Scheibler (Schlichting and Blume 1966) and subtracted the value from the total carbon concentrations.

### Statistical analyses

To analyze the nematode community structure, non-metric multidimensional scaling (NMDS) was used to check whether the composition of the nematode communities differed among plant species richness levels using the *vegdist* function in the package *vegan* (Oksanen et al. 2007) of the statistical software R (version 3.6.1, R Development Core Team, http://www.R-project.org). The NMDS was based on three dimensions, contained all 93 sampled plots and 51 identified nematode genera, and was performed for nematode abundances using Bray–Curtis dissimilarity (= Sørensen index) and for presence–absence of nematode genera based on the Jaccard index, respectively. Moreover, we fitted the nematode genera to the ordination to check which genera were most important for the distribution and thus causing significant differences along the sown plant species richness gradient using the *envfit* function in the R package *vegan* (Oksanen et al. 2007). Associated with this analysis, we also tested whether there were indicator genera, which were highly abundant only at specific levels of plant species richness using the *multipatt* function in the R package *indicspecies* (De Caceres et al. 2016).

To test whether nematode community structure differed depending on plant species richness, linear mixed-effects models were fitted using the *lmer* function in the R package *lme4* (Bates et al. 2015) with the response variables summarized in Table 2. Block and mixture identity (i.e. specific combination of plant species) were used as random effects in the models. We started with a null model with the random effects only and then added sown plant species richness (as a log-linear term) and realized plant species richness as a fixed effect, respectively. If sown plant species richness had a significant influence on a nematode variable, we checked whether this was caused by increased richness or the presence of specific plant species in mixtures using the transgressive overyielding approach (Loreau 1998): If the effects of plant species on a nematode variable are complementary, plant species mixtures will have values deviating from those expected from the “best” monoculture, which is described as transgressive overyielding (*D*_Max_; Loreau 1998). *D*_Max_ values were calculated according to the equation:

$$D_{{{\text{Max}}}} = \frac{{O_{T} - M_{{{\text{Max}}}} }}{{M_{{{\text{Max}}}} }},$$

where *O*_*T*_ is the nematode value of a given mixture, and *M*_Max_ the highest value (= “best”) among the monocultures of plant species occurring in this mixture. Based on the results of the mixed-effects model analyses, we expected that nematode diversity, bacterial feeder abundance and abundance of *r*-strategists (c–p 1 + 2 nematodes) in mixtures (positive relationships with plant species richness) should have higher values than the monoculture with the highest nematode diversity, bacterial feeder abundance, and abundance of *r*-strategists, respectively, to indicate complementarity (*D*_max_ > 0). In contrast, we expected that channel ratio and grazing pressure ratio (negative relationships with plant species richness) in the mixtures should have lower values than the monoculture with the lowest channel ratio or grazing pressure ratio, respectively, to indicate complementarity (*D*_max_ < 0). Finally, separate analyses of variance (ANOVA) with block, sown plant species richness, and mixture identity were conducted for calculated *D*_max_, to test grand means against hypothetical values (i.e., their deviation from zero), indicating whether mixtures on average showed transgressive overyielding or not. If we did not find a significant deviation of *D*_max_ from zero (= no transgressive overyielding), this relationship is probably caused by one or a few specific plant species, which also had high/low nematode values in monoculture (selection effect). Moreover, for the nematode variables that were significantly influenced by species richness, we additionally fitted mixed-effects models as explained above, but with the presence–absence of the seven target plant species as a second fixed effect in separate models (*N*_models_ = 7). This was done to assess which plant species might have caused the plant species richness effect if no transgressive overyielding was found. One exception was the analysis for scores of NMDS axis 1 and 2, which was tested for significant differences among sown plant species richness levels (categorial) with Tukey's HSD test using the function *glht* of the R package *multcomp* (Hothorn et al. 2008).

**Table 2:** Summary of mixed-effect model analyses testing the effects of sown and realized plant species richness on nematode community variables, the abundance of trophic groups, trophic group ratios, genus richness of trophic groups, nematodes in the c–p scale, and functional guild indices

	Sown plant Sr (log)				Realized plant Sr			
	*DF*	Chi^2^	*P*		*DF*	Chi^2^	*P*	
Nematode community								
Total Number per 100 g dry soil	1	0.93	0.335		1	0.59	0.443	
Genus richness	1	2.94	*0.087*	**↑**	1	2.70	*0.100*	**↑**
Shannon–Wiener diversity	1	5.00	**0.025**	**↑**	1	5.48	**0.019**	**↑**
Trophic groups – abundances								
Plant feeder	1	0.17	0.682		1	0.04	0.842	
Bacterial feeder	1	8.07	**0.004**	**↑**	1	6.24	**0.012**	**↑**
Fungal feeder	1	0.07	0.797		1	0.01	0.936	
Omnivores + predators	1	0.18	0.673		1	0.19	0.666	
Trophic groups – ratios								
Predator–prey ratio	1	0.18	0.674		1	0.08	0.778	
Channel ratio	1	5.13	**0.024**	**↓**	1	4.42	**0.036**	**↓**
Grazing pressure ratio	1	19.72	** < 0.001**	**↓**	1	15.83	** < 0.001**	**↓**
Trophic groups – genus richness								
Plant feeder	1	1.43	0.232		1	0.01	0.915	
Bacterial feeder	1	2.11	0.146		1	4.11	**0.043**	**↑**
Fungal feeder	1	0.15	0.697		1	0.25	0.620	
Omnivores + predators	1	0.72	0.395		1	0.24	0.627	
c–p scale								
c–p 1 + 2	1	5.16	**0.023**	**↑**	1	4.33	**0.038**	**↑**
c–p 3	1	0.02	0.877		1	0.08	0.784	
c–p 4	1	0.10	0.747		1	0.02	0.899	
c–p 5	1	1.95	0.162		1	4.90	**0.027**	**↑**
Functional guild indices								
Enrichment Index	1	0.28	0.595		1	0.47	0.494	
Structure Index	1	2.20	0.138		1	2.05	0.152	
Maturity Index	1	1.15	0.284		1	0.41	0.522	

Shown are degrees of freedom (DF), Chi^2^, and *P* values (*P*). Significant influences are given in bold and marginally significant influences in italics. Arrows indicate a significant increase (↑) or decrease (↓) of the measures with species richness. Note that predators and omnivores were grouped due to similar life-history strategies and that grazing pressure ratio was calculated with only seven instead of 14 monocultures (no root mass data for the second replicate)

To test whether plant species richness effects on the nematode community can be explained by a change in resource quantity or quality, we applied piecewise structural equation modeling (SEM) for nematode community indices (total number of nematodes, genus richness, nematode diversity, scores of NMDS axis 2 [nematodes were spread along the species richness gradient on NMDS axis 2]), for the abundance of trophic groups, for genus richness of trophic groups, and for the c–p scale in separate models (due to similar life-history strategies, predators and omnivores were grouped to keep the SEM clearly structured). We started with an initial model for all models containing the possible causal drivers of nematode variables and their interrelationships based on current knowledge. We used all measured plant and soil variables, except root length density (strong correlation with root mass) and soil nitrogen concentration (strong positive correlation with soil organic carbon). Piecewise SEMs were run with 86 plant communities (no root data available for the second replicate of the monocultures) and were based on linear mixed-effects models using the function *psem* of the R package *piecewiseSEM* (Lefcheck 2016) for accounting for block and mixture identity as random effects. Model fit was assessed using Fisher's C statistic, where *P* > 0.05 indicates that the data are well represented by the model. Before linear mixed-effects model and SEM analyses, variables were transformed to meet the assumptions of normality and variance homogeneity: sown plant species richness, the abundance of bacterial feeders, fungal feeders and omnivores + predators, predator–prey ratio, c–p 1 + 2 nematodes, and c–p 4 nematodes were log-transformed; genus richness of fungal feeders and nematodes in c–p 3 and 4 were square root-transformed.

## Results

### Nematode community composition

We extracted on average 1852.4 ± 724.2 (SD) nematode individuals 100 g^−1^ dry soil (highest number: 3604.9 nematodes g^−1^ dry soil; lowest number: 538.7 nematodes g^−1^ dry soil). We identified 51 different genera in total (Table S2), whereas most genera belonged to the plant feeder and bacterial feeder groups (18 genera, respectively). Fungal feeders, omnivores, and predators were less diverse (5, 7, and 3 genera). The most abundant trophic group was the plant-feeding group with 63.6% of total number of identified nematodes. Bacterial feeders, fungal feeders, and omnivores had similar proportion with ~ 10% (12.3%, 13.0%, and 8.9%), while the predatory group was the one with the lowest percentage (2.3%).

### Plant species richness effects on nematode community composition and diversity

**Fig. 1:**
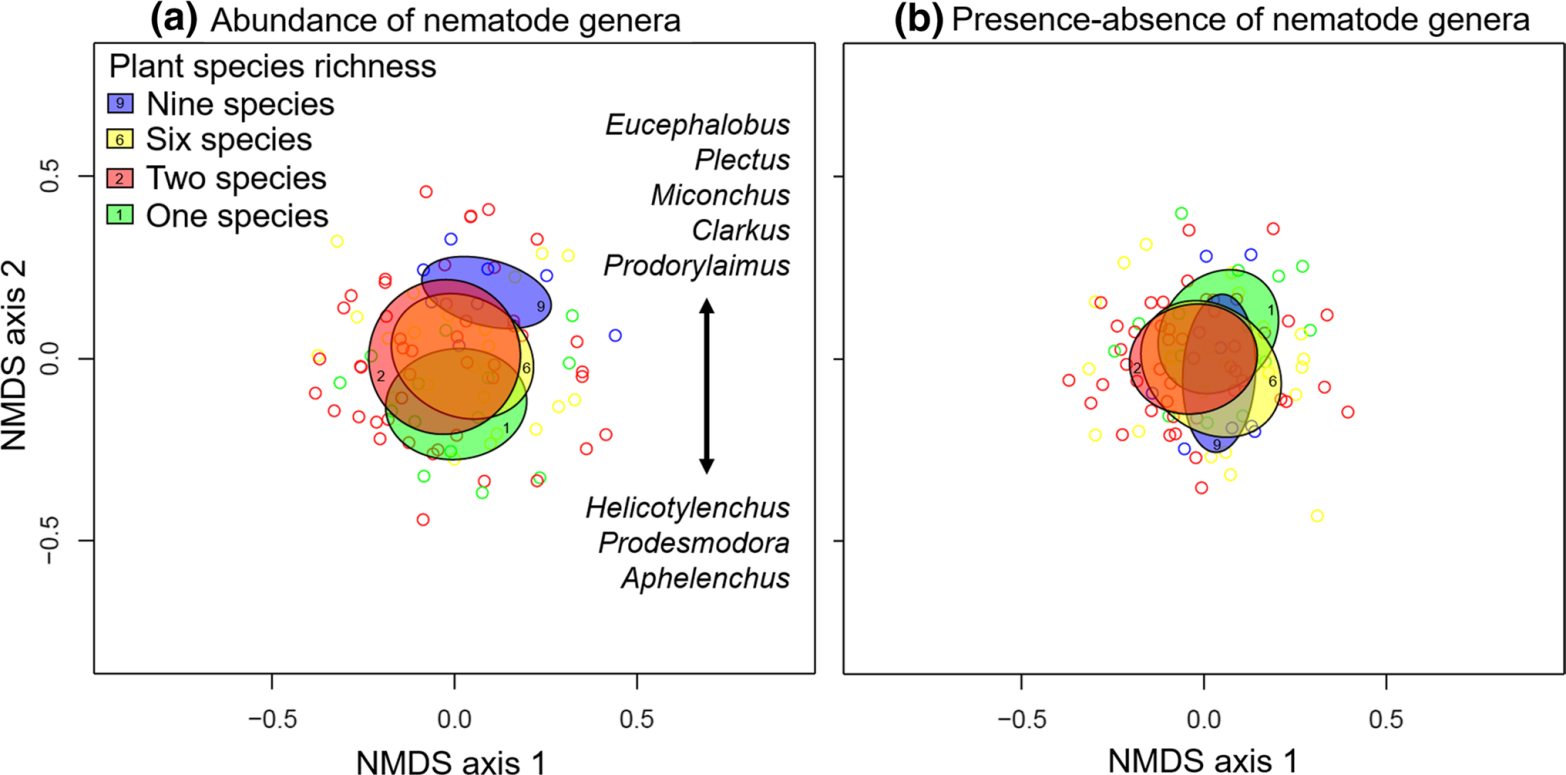
Summary of non-metric multidimensional scaling (NMDS), abundance based on Bray–Curtis dissimilarity (**a**) and presence–absence based on Jaccard index (**b**) of 51 nematode genera identified in 93 plant communities. Circles indicate the plant communities differing in sown plant species richness, and the ellipses indicate the standard deviation of point scores for each plant species richness level. Arrow in NMDS for abundance (**a**) indicates which nematode genera were most important for the distribution of circles and ellipses along the sown plant species richness gradient

NMDS analysis based on nematode abundances revealed that nematode communities were spread along the sown plant species richness gradient on the NMDS axis 2 (Fig. 1a). The nematode composition differed significantly between monocultures and nine-species communities (Fig. 1a; Table S3), while two- and six-species communities were located between these extremes of the diversity gradient (Fig. 1a). We found several genera, which were significantly related to the second dimension of the ordination (*P* < 0.01; Table S2). Significant genera, which had high negative loadings (< − 0.4) for NMDS axis 2 were *Aphelenchus* (FF2 [number indicates c–p scale]), *Prodesmodora* (BF3), and *Helicotylenchus* (PF3), and separating the monocultures, while the genera with high positive loadings (< 0.4) were *Eucephalobus* (BF2), *Plectus* (BF2), *Miconchus* (Pr4), *Clarkus* (Pr4), and *Prodorylaimus* (Om5) separating the nine-species communities (Table S2). NMDS analysis based on the presence-absence of genera revealed no significant differences among nematode communities along the plant species richness gradient (Fig. 1b). Indicator analysis showed that the genera *Protorhabditis* (BF1), *Eucephalobus* (BF2), *Wilsonema* (BF2), *Lelenchus* (PF2), *Prodorylaimus* (Om5), and *Aporcelaimellus* (Om5) were highly abundant genera in the nine-species communities, while other plant species richness-levels showed no significant indicator genera.

**Fig. 2:**
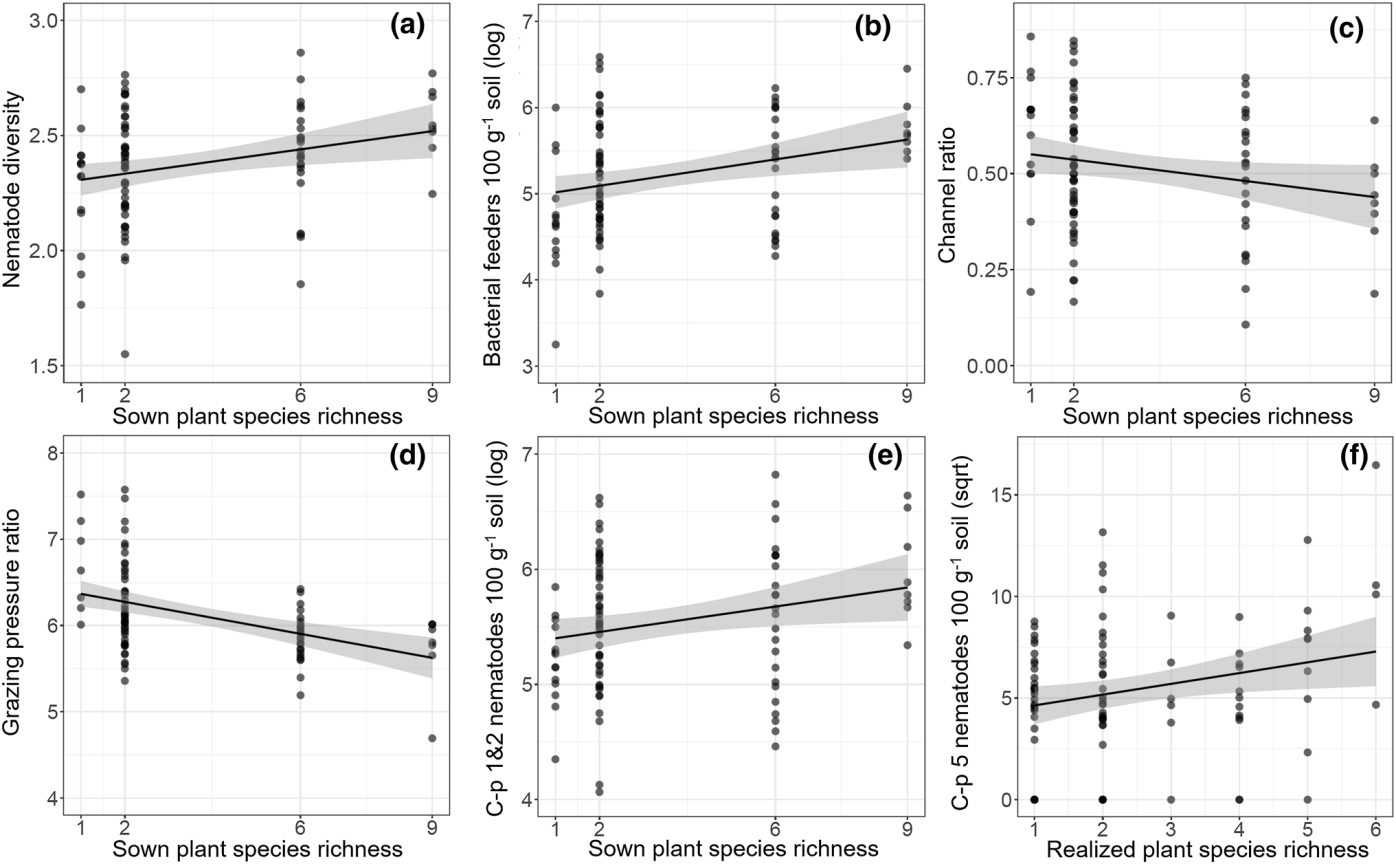
Relationships between plant species richness and nematode diversity (**a**), number of bacterial feeders 100 g^−1^ dry soil (log-transformed; **b**), channel ratio (= FF (FF + BF)^−1^; **c**), grazing pressure ratio (= log (PF root mass^−1^); **d**), number of c–p 1 and 2 nematodes 100 g^−1^ dry soil (log-transformed; **e**), and number of c–p 5 nematodes 100 g^−1^ dry soil (square root-transformed; **f**). Each circle represents a plant community, lines (± SE) indicate significant relationships of linear mixed-effects models (*P* < 0.05; Table 2)

In general, we found similar mixed-effects model results for sown plant species richness and realized plant species richness (Table 2). Nematode diversity significantly increased with plant species richness (Fig. 2a), while the total number of nematodes and genus richness showed no significant relationship with plant species richness (Table 2). Nematode diversity in plant mixtures was not significantly higher than nematode diversity of the “best” monoculture (no transgressive overyielding; *F*_1,43_ = 0.165, *P* = 0.687), but we detected two grass species, *A. elatius* and *D. glomerata*, which positively influenced nematode diversity (*A. elatius*: Chi^2^ = 6.89, *P* = 0.009; *D. glomerata*: Chi^2^ = 5.58, *P* = 0.018; monoculture with highest diversity: *A. elatius*).

**Fig. 3:**
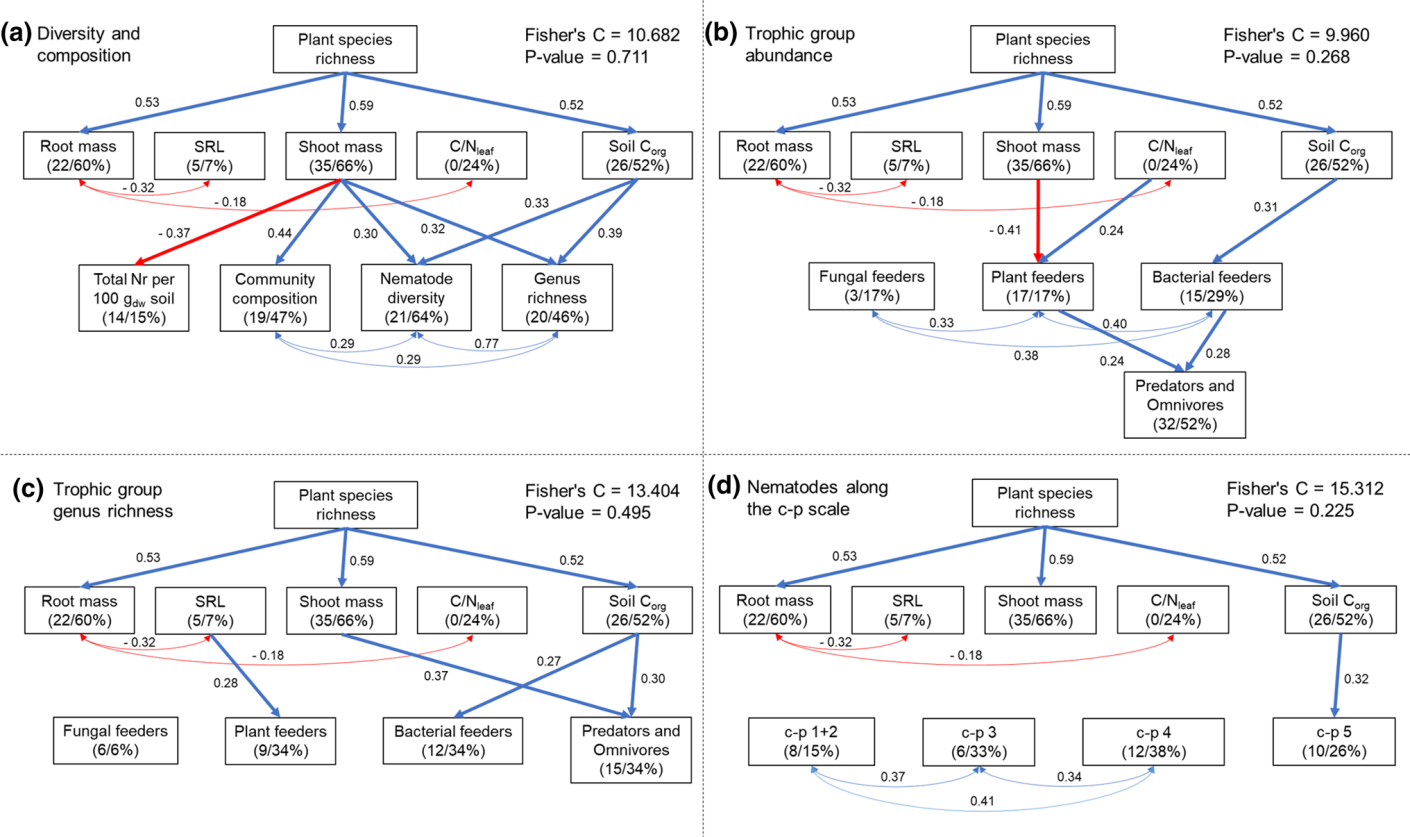
Piecewise structural equation models (SEM) exploring the effect of sown plant species richness, root mass, specific root length (SRL), shoot mass, *C*/*N* ratio of leaves (*C*/*N*_leaf_), and soil organic carbon concentrations (soil *C*_org_) on nematode diversity and composition (**a**), trophic group abundance (**b**), trophic group genus richness (**c**), and nematodes along the c–p scale (**d**). Arrows represent significant unidirectional relationships among variables (*P* < 0.05), while blue arrows indicate positive relationships and red arrows indicate negative relationships. Double-headed arrows show correlated errors. Standardized parameter estimates are given next to the arrows. Marginal *R*^2^ (based on fixed effects only) and conditional *R*^2^ (based on random and fixed effects) for component models with significant relationships are given in brackets below the respective response variable

Piecewise SEM revealed that plant species richness positively influenced most of the measured plant and soil variables; except specific root length, which was marginally significantly and negatively related to plant species richness, as well as *C*/*N*_leaf_ ratio, which showed no relationship with plant species richness (Fig. 3). The positive effects of plant species richness on nematode diversity were explained via increased shoot mass and soil organic carbon concentrations with higher plant species richness, which was also shown for genus richness (Fig. 3a). Moreover, shoot mass was positively related to nematode community composition (scores of NMDS axis 2) and negatively associated with the total number of nematodes (Fig. 3a).

### Plant species richness effects on trophic groups and their ratios

Linear mixed-effect model analysis revealed that the abundance and genus richness of bacterial feeders were positively related with plant species richness (genus richness only with realized plant species richness; Table 2; Fig. 2b), while channel ratio and grazing pressure ratio were negatively related with plant diversity (Table 2; Fig. 2c, d). Bacterial feeder abundance in mixtures was higher than expected from monocultures (transgressive overyielding; *F*_1,42_ = 8.91, *P* = 0.005), while grazing pressure ratio in mixtures was lower than expected from monocultures (*F*_1,43_ = 13.41, *P* < 0.001). In addition, plots with the grass *A. elatius* and the legume *T. repens* showed lower grazing pressure than communities without these plant species (*A. elatius*: Chi^2^ = 13.71, *P* < 0.001; *T. repens*: Chi^2^ = 4.79, *P* = 0.029).

Piecewise SEM for trophic group abundances revealed that the positive influence of plant species richness on the number of bacterial feeders was induced via increased soil organic carbon concentrations (Fig. 3b). Further, we detected that the number of plant feeders was positively affected by *C*/*N*_leaf_ ratio and negatively by shoot mass (Fig. 3b). The number of omnivores and predators were positively influenced by the number of plant feeders and bacterial feeders (Fig. 3b). Piecewise SEM for genus richness within trophic groups showed that plant feeder richness was positively affected by specific root length and bacterial feeder richness by soil organic carbon concentrations (Fig. 3c). Genus richness of omnivores and predators was also positively influenced by soil organic carbon and additionally by shoot mass (Fig. 3c).

### Plant species richness effects on *r*- and *K*-strategists and functional guild indices

We found a positive relationship between plant species richness and abundance of c–p 1 and 2 nematodes (abundance in mixtures was higher than expected from monocultures, *F*_1,43_ = 28.51, *P* < 0.001) and between plant species richness and abundance of c–p 5 nematodes (only with realized plant species richness; Table 2; Fig. 2e, f), while other c–p groups were not significantly influenced. Piecewise SEM indicated that the positive influence of plant species richness on c–p 5 nematodes was mediated via enhanced soil organic carbon concentration (Fig. 3d). We found no significant influence of plant diversity on functional guild indices (SI, EI, MI).

## Discussion

### Plant species richness effects on nematode community composition and diversity

We generally found that nematode community composition (NMDS for abundance) and nematode diversity differed among the plant species richness levels, which is in line with recent studies (Cortois et al. 2017; De Deyn et al. 2004; Eisenhauer et al. 2011; Guerrero‐Ramírez et al. 2019). Interestingly, the ellipses in the NMDS, which indicate the standard deviation (SD) of point scores for each plant species richness level, did not strongly differ in their size indicating similar variability in community composition. Several microcosm experiments (Niu et al. 2019; Wardle et al. 2003) and field studies (De Deyn et al. 2004; Viketoft et al. 2005) have shown that plant species strongly differ in their effects on nematode community composition. Thus, we expected that monocultures show the highest variation (i.e. largest ellipse); however, we found that monocultures showed a lower within-variation than two- and six-species communities (area_mono_ = 0.13; area_two_ = 0.20; area_six_ = 0.15; area_nine_ = 0.10, see Fig. S1 for calculation). A possible explanation could be that plant species in monoculture were exposed to a similar selection environment, i.e. the accumulation of soil-borne pathogens favors individuals, which are able to defend and persist (Eisenhauer et al. 2019). This may lead to similar trade-offs in “functioning” of plant species in monoculture, e.g., higher investment in chemical and morphological defense traits, which in turn could similarly shape the nematode community composition. Thus, short-term effects of single plant species on nematode communities could be diluted over time. The NMDS analysis based on presence–absence did not reveal any significant differences among plant species richness levels, indicating that most nematode genera were present in all plant species richness levels. Both NMDS results together show that compositional differences along the plant species richness gradient were mainly caused by increased or decreased abundance of genera but not by specific genera, which only occur under certain plant species richness levels. This is also supported by the indicator analysis which revealed no indicator genera, except for the nine-species plots. One possible reason for the detection of indicator genera in the nine-species plots could be that these communities provide the highest resource quality or quantity, which enables the existence of specific genera (for example the large *K*-strategists *Prodorylaimus* and *Aporcelaimellus;* see discussion on bacterial feeders and omnivores). However, we cannot exclude that we found indicator genera because the nine-species communities were eight identical replicates of the same plant species composition, which caused a low within-variation (smallest ellipse) and thus higher chance to detect indicator genera.

Although we found a positive influence of plant species richness on nematode diversity, we did not detect higher values in mixtures than expected from monocultures. Instead, the two most-productive plant species of the Dominance Experiment, *A. elatius* and *D. glomerata* (Roscher et al. 2007) showed significant positive effect on nematode diversity next to species richness. This is in line with the SEM results showing that positive effects of plant species richness on nematode diversity, genus richness, and composition were induced via increased shoot mass. An additional pathway was shown via increased soil organic carbon concentrations. These results indicate that nematode diversity and composition depend mainly on resource quantity and that high-productive, dominant plant species (i.e. *A. elatius* and *D. glomerata* in our study) play an important role for the diversity of nematodes, which is in line with a recent study (Wang et al. 2019).

The increase of soil organic carbon with plant species richness is in line with recent studies showing that higher plant diversity leads to an increase of root exudates, which in turn increases the activity and growth of soil biota (Eisenhauer et al. 2017; Lange et al. 2015; Prommer et al. 2020). Hence, we conclude that positive plant diversity effects were induced via increased microbial biomass, which enhances the diversity and composition of nematode communities (Eisenhauer et al. 2013), particularly bacterial feeders and omnivores (as shown in the SEM for trophic group genus richness).

In contrast to our expectations and previous findings (Eisenhauer et al. 2013), we did not find any influence of root mass on nematode diversity or composition but a strong influence of shoot mass. The lack of the effect was also present when we excluded shoot mass from the SEM analysis (not shown). We can only speculate about the reasons, but it is plausible that more shoot mass and denser vegetation caused a reduction of evaporation and increased topsoil moisture (0–10 cm; Fischer et al. 2018; Wright et al. 2015). Further, higher shoot mass could have also buffered negative impacts by drought, e.g., due to shading, leading to more stable soil moisture over the years (Wright et al. 2017). Indeed, Lange et al. (2014) showed that denser vegetation promotes higher soil microbial activity via increased soil moisture in the Jena Experiment. Thus, we conclude that nematode diversity could be increased by positive microclimatic effects due to more stable and beneficial soil moisture, which enables, for example, the establishment of large *K*-strategist, and/or by positive effects via increased microbial activity (Lange et al. 2015) promoting the establishment of different microbial-feeding and omnivorous genera (Yan et al. 2018). In addition, higher shoot mass could indicate elevated levels of photosynthates released into the rhizosphere, which could boost bottom–up effects on microbivores (Eisenhauer et al. 2017). The positive impact of shoot mass and dominant grass species is probably the reason why we did not find higher nematode diversity in mixtures than expected from monocultures (no transgressive overyielding) because monocultures of dominant plant species (in particular *A. elatius*) showed high aboveground biomass production and thus also high nematode diversity.

Furthermore, SEM results indicated that shoot mass has a negative impact on the total number of nematodes, which is explainable by a decrease of plant feeders with increasing shoot mass (as shown in the SEM for trophic group abundances). Plant feeders had the most considerable portion of the total number of nematodes per plot, ranging between 40% and 90%, while other trophic groups had a lower amount between 1% and 30%, respectively. It is plausible that plant feeders experienced more control by predators and omnivores at higher plant diversity (Barnes et al. 2020) leading to an elevated top–down pressure and thus lower number of plant-feeding nematodes (see discussion on plant feeders).

### Plant species richness effects on trophic groups, their ratios, and *r*- and *K*-strategists

We found significant higher abundance of bacterial feeders in mixtures than in the “best” monoculture, indicating a strong positive influence of plant species richness on this trophic group. SEM revealed that the positive diversity effect was caused by increased soil organic carbon. Guenay et al. (2013) found in the Dominance Experiment transgressive overyielding for soil microbial biomass, suggesting that bacterial feeders benefitted from the higher amounts of resources with increasing plant species richness (Eisenhauer et al. 2013). A possible mechanism underlying these relations could be that higher plant species richness leads to a higher amount and diversity of root exudates, which positively influence soil microbial biomass (Eisenhauer et al. 2017; Prommer et al. 2020) and thus abundance and richness of bacterivorous nematodes. We, therefore, conclude that bacterial feeders were mainly dependent on plant diversity-induced effects of resource quantity. This is in line with the results found for the c–p 1 + 2 group, as 13 out of 16 genera within this group were bacterivorous.

In contrast to bacterial feeders, fungal feeders did not respond significantly to increasing plant species richness. The increase of bacterial feeders and the stable number of fungal feeders with increasing plant diversity led to a change from more fungal-dominated systems to more bacteria-dominated systems, indicated by a significant decrease of the channel ratio with increasing species richness. Our results contradict previous findings showing that plant communities become more fungal-dominated with increasing plant diversity (Bennett et al. 2020; Eisenhauer et al. 2011, 2017); however, there was one significant difference between previous work and our study. Plant communities in our study were dominated by grasses (relative biomass production of grasses in two-species plots was 71% ± 6% (SE) and increased up to 89% ± 3% in nine-species plots), while in the other studies, there was a more balanced combination of herbs, legumes and grasses in mixtures (Marquard et al. 2009). Plant functional group identity and diversity differently influence microbial-feeding nematodes (Cortois et al. 2017; Viketoft et al. 2005), so that the different composition of plant mixtures probably explain the contrasting results, but this hypothesis needs further testing.

We found no direct impact of plant species richness on omnivores and predators; but SEM analysis showed that the abundance of omnivores and predators was positively influenced by the abundance of bacterial and plant feeders. Several studies support our results by showing that omnivores and predators were positively impacted by other trophic groups of nematodes functioning as a food source (Cortois et al. 2017; Eisenhauer et al. 2013; Khan and Kim 2007). Furthermore, SEM results showed that genus richness of omnivores and predators was enhanced via increased shoot mass and soil organic carbon, indicating an indirect positive influence of plant species richness. As explained for nematode diversity, shoot mass, and thus denser vegetation can buffer adverse drought effects, which leads to a more stable microenvironment and enables a higher richness of large *K*-strategic omnivores and predators, as shown in Yan et al. (2018). In addition to this, higher soil organic carbon could enhance the richness of omnivores and predators, either due to higher microbial biomass (as a resource for omnivores) and/or higher abundance of bacterial-feeding nematodes (as a resource for omnivores and predators). In conclusion, results of the present study indicate that omnivores and predators were dependent on plant diversity-induced effects of resource quantity (and microenvironmental conditions), similar to bacterial feeders. This conclusion is supported by the results found for c–p 5 nematodes (positive relationship between abundance and realized plant species richness; SEM), since all genera belonging to the c–p 5 group were omnivorous.

Interestingly, plant species richness did not influence plant feeder abundance or richness, but grazing pressure ratio significantly decreased with increasing plant diversity. This supports previous findings indicating a higher accumulation of pathogens in low-diversity than in high-diversity communities (Cortois et al. 2017; Kulmatiski et al. 2012; Schnitzer et al. 2011). This dilution effect in species-rich communities is a possible explanation contributing to the positive diversity–productivity relationship found in this study and many biodiversity experiments (Cardinale et al. 2007; Marquard et al. 2009; Roscher et al. 2007). We did not find a significant influence of the plant species *A. elatius* and *T. repens* on the abundance of plant feeders, but a positive impact on grazing pressure. Thus, we think that these dilution effects were caused by the promotion of root mass production (bottom–up effect) and not by suppressing plant feeders. The grass *A. elatius* directly increased root mass due to general high productivity (as dominant species in the Jena Experiment), while it is likely that the legume *T. repens* indirectly increased the root mass production of the whole community due to legume-specific interactions with rhizobacteria increasing the soil nitrogen concentrations (Bessler et al. 2012).

Piecewise SEM analysis showed that the abundance of plant feeders increased with a higher *C*/*N*_leaf_ ratio and genus richness of plant feeders with higher specific root length. These results indicate that plant feeders, in contrast to other trophic groups, were controlled by resource quality. The positive relationship between plant feeders and aboveground plant *C*/*N* ratios was also found in the Jena Experiment in previous studies for nematodes (Cortois et al. 2017) and aboveground arthropods (Ebeling et al. 2014). Both studies stated that this is an unexpected result because the abundance of herbivores should increase with the higher nutritional quality (i.e. lower *C*/*N*) of the food source (Cebrian et al. 2009). Further, they argued that supporting tissues, such as plant stems, have higher *C*/*N* ratios and that such tissues increased with increasing plant species richness (Roscher et al. 2007), which probably explains the unexpected relationship. In our study, we determined the *C*/*N* ratio of leaves, not of shoot mass, but still found this positive relationship. Perhaps, there is an undiscovered influence and/or correlated variables, which should be investigated in more detail in the future. The positive relationship between specific root length and plant feeder richness indicates that plant feeders depend on a higher fineness of roots, which is to some extent in line with previous studies. Otfinowski and Coffey (2020) showed that higher SRL increased the abundance of plant-feeding nematodes, and Cortois et al. (2016) showed that plants with higher SRL more strongly suffer from soil-borne pathogens. We assume that lower tissue density (Otfinowski and Coffey 2020) or higher nutritional quality of fine roots (Gordon and Jackson 2000) enhanced herbivory attractivity and thus the richness of plant feeders. Next to the resource quality impacts on plant feeders, we also detected shoot mass as a proxy for resource quantity influencing plant feeder abundance. Interestingly, this relationship was not positive, as expected, but negative. This indicates that plant feeders were not controlled by higher food quantity, but probably by a more substantial top–down control by predators and omnivores. As described above, a higher shoot mass may cause a lower vulnerability to drought, enhancing the abundance and richness of omnivores and predators. This, in turn, could increase the top–down control (Barnes et al. 2020), leading to a lower plant feeder abundance in communities with more stable soil moisture over the years (Franco et al. 2020; Wilschut and Geisen 2021).

## Conclusion

In the present study, we showed that nematode communities in 15-year-old plant communities significantly changed along the plant species richness gradient, caused by strong bottom–up effects on almost all trophic groups (except fungal feeders). We found evidence that bacterial feeders, omnivores, and predators were controlled mainly by resource quantity, leading to a higher abundance and richness of these trophic groups in species-rich plant communities. Contrary to this, plant feeders were primarily controlled by resource quality and showed a higher accumulation in species-poor plant communities (likely causing higher top-down pressure on plants). Our results confirm the assumption by Eisenhauer et al. (2012) that soil mutualists, such as bacterivores, predators, and omnivores (Wilschut and Geisen 2021), accumulate in high-diversity plant communities over time, while in low-diversity plant communities there is an accumulation of soil-borne plant antagonists, such as plant-feeding nematodes. The opposing accumulation of mutualistic and antagonistic nematodes can explain, at least in part, the positive strengthening relationship between plant productivity and diversity over time, which was found in many long-term biodiversity experiments (Guerrero-Ramirez et al. 2017; Reich et al. 2012; Tilman et al. 2006). The next promising steps forward would be the set-up of plant–soil feedback experiments to verify soil nematodes' contribution to plant diversity–productivity relationships (Guerrero‐Ramírez et al. 2019) and the direct manipulation of nematode communities to steer plant community functioning.

## Supplementary Information

Below is the link to the electronic supplementary material.

Supplementary file1 (DOCX 54 kb)

## Data Availability

The data reported in this paper have been deposited in BExIS, which can be publicly accessed at https://jexis.uni-jena.de/ddm/data/Showdata/160 and https://jexis.uni-jena.de/ddm/data/Showdata/161.
