# Supplementary material for: Effects of plant species diversity on nematode community composition and diversity in a long-term biodiversity experiment
Source: Oecologia. 2021 Jun 6;197(2):297–311. doi: 10.1007/s00442-021-04956-1 (PMC8505370; doi:10.1007/s00442-021-04956-1)
Supplement: Supplementary file 1 — Supplementary file1 (DOCX 54 kb) [file 442_2021_4956_MOESM1_ESM.docx]

**Supplementary material**

Article: Effects of plant species diversity on nematode community composition and diversity in a long-term biodiversity experiment

Authors: Peter Dietrich*, Simone Cesarz, Tao Liu, Christiane Roscher and Nico Eisenhauer

*corresponding author: peter.dietrich@idiv.de

**Tables**

**Table S1** Summary of the two-species communities, which were used for the soil and plant sampling.

|  | Number of used replicates containing: | | | | | | | |
| --- | --- | --- | --- | --- | --- | --- | --- | --- |
|  | *A. pratensis* | *D. glomerata* | *P. trivialis* | *G. pratense* | *T. pratense* | *T. repens* | *A. sylvestris* | *P. pratense* |
| *A. elatius* | 2 | 2 | 2 | 2 | 1* | 1* | 1* | 1* |
| *A. pratensis* | - | 2 | 2 | 2 | 1* | 1* | 1* | 1* |
| *D. glomerata* |  | - | 2 | 2 | 1* | 1* | 1* | 1* |
| *P. trivialis* |  |  | - | 2 | 2 | 2 | 1* | 1* |
| *G. pratense* |  |  |  | - | 2 | 2 | 0° | 0° |
| *T. pratense* |  |  |  |  | - | 1* | 2 | 1* |
| *T. repens* |  |  |  |  |  | - | 0° | 1* |

*only one replicated community was sampled due to low abundance of *T. pratense*, *T. repens*, *A. sylvestris* or *P. pratense*

°in these plots the target species went extinct; therefore, we did not take samples

**Table S2** Summary of the fitting of nematode genera to the NMDS ordination. Shown are nematode genera, their classification into trophic groups and c-p scale, scores for NMDS axis 1 and 2 (Axis 1; Axis 2), coefficients of determination (R^2^) and P-values (P). Significant relationships are given in bold, marginally significant relationships in italics.

| Nematode genus | Trophic group | C-p scale | NMDS | | | |
| --- | --- | --- | --- | --- | --- | --- |
|  |  |  | Axis 1 | Axis 2 | R^2^ | P |
| *Brevibucca* | Bacterial feeder | 1 | -0.995 | -0.100 | 0.02 | 0.528 |
| *Protorhabditis* | Bacterial feeder | 1 | -0.612 | 0.791 | 0.14 | **0.003** |
| *Rhabditis* | Bacterial feeder | 1 | -0.859 | -0.512 | 0.03 | 0.214 |
| *Acrobeles* | Bacterial feeder | 2 | 0.235 | -0.972 | 0.01 | 0.618 |
| *Acrobeloides* | Bacterial feeder | 2 | -0.632 | 0.775 | 0.05 | 0.122 |
| *Cephalobus* | Bacterial feeder | 2 | 0.235 | 0.972 | 0.04 | 0.167 |
| *Cervidellus* | Bacterial feeder | 2 | -0.182 | 0.983 | 0.02 | 0.502 |
| *Eucephalobus* | Bacterial feeder | 2 | -0.224 | 0.975 | 0.41 | **0.001** |
| *Eumonhystera* | Bacterial feeder | 2 | 0.709 | 0.705 | 0.05 | *0.074* |
| *Heterocephalobus* | Bacterial feeder | 2 | 0.973 | -0.229 | 0.08 | **0.014** |
| *Monhystera* | Bacterial feeder | 2 | 0.879 | -0.477 | 0.01 | 0.565 |
| *Plectus* | Bacterial feeder | 2 | -0.097 | 0.995 | 0.13 | **0.001** |
| *Wilsonema* | Bacterial feeder | 2 | 0.544 | 0.839 | 0.04 | 0.105 |
| *Prismatolaimus* | Bacterial feeder | 3 | 0.378 | 0.926 | 0.09 | **0.020** |
| *Prodesmodora* | Bacterial feeder | 3 | -0.601 | -0.799 | 0.27 | **0.001** |
| *Rhabdolaimus* | Bacterial feeder | 3 | 0.972 | 0.233 | 0.05 | *0.084* |
| *Teratocephalus* | Bacterial feeder | 3 | 0.097 | 0.995 | 0.04 | 0.124 |
| *Alaimus* | Bacterial feeder | 4 | -0.943 | 0.332 | 0.20 | **0.001** |
| *Aphelenchoides* | Fungal feeder | 2 | 0.723 | 0.691 | 0.06 | *0.064* |
| *Aphelenchus* | Fungal feeder | 2 | -0.904 | -0.428 | 0.15 | **0.001** |
| *Filenchus* | Fungal feeder | 2 | 0.997 | 0.073 | 0.05 | 0.106 |
| *Diphtherophora* | Fungal feeder | 3 | 0.952 | -0.305 | 0.00 | 0.967 |
| *Tylencholaimus* | Fungal feeder | 4 | -0.911 | -0.411 | 0.05 | *0.090* |
| *Clarkus* | Predators | 4 | 0.707 | 0.708 | 0.21 | **0.001** |
| *Miconchus* | Predators | 4 | 0.293 | 0.956 | 0.15 | **0.001** |
| *Mylonchulus* | Predators | 4 | -0.987 | 0.163 | 0.13 | **0.002** |
| *Eudorylaimus* | Omnivores | 4 | -0.840 | -0.542 | 0.03 | 0.322 |
| *Microdorylaimus* | Omnivores | 4 | -0.019 | 1.000 | 0.04 | 0.164 |
| *Thornia* | Omnivores | 4 | -0.577 | 0.817 | 0.00 | 0.916 |
| *Aporcelaimellus* | Omnivores | 5 | 0.695 | 0.719 | 0.04 | 0.195 |
| *Discolaimus* | Omnivores | 5 | -0.999 | -0.041 | 0.14 | **0.001** |
| *Mesodorylaimus* | Omnivores | 5 | -0.992 | -0.129 | 0.02 | 0.343 |
| *Prodorylaimus* | Omnivores | 5 | 0.911 | 0.413 | 0.19 | **0.001** |
| *Aglenchus* | Plant feeders | 2 | -0.383 | -0.924 | 0.07 | **0.048** |
| *Boleodorus* | Plant feeders | 2 | 0.996 | -0.092 | 0.10 | **0.008** |
| *Coslenchus* | Plant feeders | 2 | 0.213 | 0.977 | 0.07 | **0.026** |
| *Ecphyadophora* | Plant feeders | 2 | 0.918 | -0.397 | 0.00 | 0.966 |
| *Lelenchus* | Plant feeders | 2 | -0.183 | 0.983 | 0.02 | 0.448 |
| *Malenchus* | Plant feeders | 2 | -0.995 | -0.100 | 0.02 | 0.528 |
| *Paratylenchus* | Plant feeders | 2 | -0.398 | 0.917 | 0.07 | **0.042** |
| *Psilenchus* | Plant feeders | 2 | -0.990 | 0.143 | 0.08 | **0.020** |
| *Tylenchidae* | Plant feeders | 2 | -0.173 | 0.985 | 0.06 | **0.011** |
| *Tylenchus* | Plant feeders | 2 | -0.964 | 0.267 | 0.08 | **0.016** |
| *Bitylenchus* | Plant feeders | 3 | -0.960 | 0.279 | 0.08 | **0.026** |
| *Criconemoides* | Plant feeders | 3 | -0.757 | -0.654 | 0.00 | 0.874 |
| *Helicotylenchus* | Plant feeders | 3 | -0.581 | -0.814 | 0.16 | **0.001** |
| *Hoplotylus* | Plant feeders | 3 | -0.393 | 0.919 | 0.01 | 0.645 |
| *Macroposthonia* | Plant feeders | 3 | 0.770 | -0.638 | 0.06 | *0.056* |
| *Ogma* | Plant feeders | 3 | 0.097 | 0.995 | 0.04 | 0.124 |
| *Pratylenchus* | Plant feeders | 3 | 0.976 | 0.219 | 0.10 | **0.003** |
| *Trophurus* | Plant feeders | 3 | 0.976 | -0.218 | 0.03 | 0.330 |

**Table S3** Summary of mixed-effect model analysis testing the effects of sown plant species richness on scores of NMDS axis 1 and 2 as well as Tukey´s HSD analysis testing for differences in scores of NMDS axis 1 and 2 among plant species richness levels (categorial). Shown are Chi^2^ and P-values (P) for mixed-effect model analysis and Z-values and P-values (P) for Tukey´s HSD test. Significant differences are given in bold.

|  | NMDS axis 1 | | NMDS axis 2 | |
| --- | --- | --- | --- | --- |
| Mixed-effect analysis: | Chi^2^ | P | Chi^2^ | P |
| Plant species richness | 1.56 | 0.212 | 4.71 | **0.030** |
| Tukey´s HSD | Z | P | Z | P |
| 1 vs. 2 | -0.46 | 0.966 | -2.24 | 0.105 |
| 1 vs. 6 | 0.29 | 0.991 | -2.15 | 0.128 |
| 1 vs. 9 | 1.02 | 0.730 | -3.07 | **0.011** |
| 2 vs. 6 | -0.95 | 0.771 | 0.18 | 0.998 |
| 2 vs. 9 | -1.55 | 0.397 | 1.99 | 0.180 |
| 6 vs. 9 | -0.87 | 0.815 | 1.82 | 0.252 |

|  | Sown plant Sr (log) | | |  | Realized plant Sr | | |
| --- | --- | --- | --- | --- | --- | --- | --- |
|  | DF | Chi^2^ | P |  | DF | Chi^2^ | P |
| Abundances |  |  |  |  |  |  |  |
| Predators (sqrt) | 1 | 0.01 | 0.906 |  | 1 | 0.85 | 0.357 |
| Omnivores (sqrt) | 1 | 1.04 | 0.307 |  | 1 | 1.69 | 0.193 |
| Genus richness |  |  |  |  |  |  |  |
| Omnivores (sqrt) | 1 | 1.04 | 0.307 |  | 1 | 1.69 | 0.193 |

**Table S4** Summary of mixed-effect model analysis testing the effects of sown and realized plant species richness on the abundance of omnivores and predators, and genus richness of omnivores. Shown are degrees of freedom (DF), Chi^2^, and P-values (P). Note that predators' genus richness was not analyzed because of a low number of different genera (N=3).

**Figures**


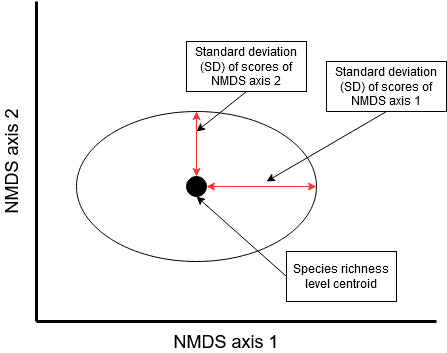


**Figure S1** Shown is NMDS ellipse, which indicates the standard deviation (SD) of point scores for one plant species richness level. The black dot indicates the centroid of the ellipse, while the red arrows indicate the standard deviation of scores of NMDS axis 1 and 2, respectively. The area of each NMDS ellipses was calculated after the following equation: $\text{Area}_{\text{ellipse}}\text{= π ×}{\text{ }\text{SD}}_{\text{NMDS axis 1}}\text{×}{\text{ }\text{SD}}_{\text{NMDS axis 2}}$
